# The Impact of Structural and Meso-Level Factors on Caregiver Coping Abilities When Supporting a Child with Cancer: A Qualitative Study

**DOI:** 10.3390/ijerph21070907

**Published:** 2024-07-11

**Authors:** Vivian Christensen, Melissa Varnum, Kellee Parker, Lai Hin Kimi Chan, Lauren Saxton, Erika Cottrell

**Affiliations:** 1 Oregon Clinical and Translational Research Institute (OCTRI), Oregon Health and Science University, 3181 SW Sam Jackson Park Road, Portland, OR 97239, USA; 2 Department of Pediatrics, Division of Pediatric Hematology and Oncology, University of Utah, 81 N Mario Capecchi Dr., Salt Lake City, UT 84113, USA; 3 Department of Family Medicine, University of California Davis, 4860 Y Street Suite 1600, Sacramento, CA 95817, USA; 4 OCHIN, Inc., 1881 SW Naito Pkwy, Portland, OR 97201, USA

**Keywords:** childhood cancer, caregivers, coping, qualitative research

## Abstract

Family caregivers of children diagnosed with cancer often experience periods of significant stress. We provide an in-depth examination of the impacts of structural (health care and leave policies) and meso-level (organizations and communities/social networks) factors on caregiver coping during childhood cancer treatment. We conducted a secondary analysis of a comprehensive qualitative dataset examining the impacts of structural and meso-level factors on caregiver coping from in-depth, semi-structured interviews with 49 caregivers representing 38 unique cases of childhood cancer. Using a modified grounded theory approach, transcripts were analyzed using inductive thematic analysis. Caregivers experienced multiple and often intersecting structural and meso-level factors, both facilitating and impeding their ability to cope during their child’s cancer treatment. Our analysis revealed the following themes: having few out-of-pocket medical expenses, access to paid time off from employment, and support from one’s health system, organizations, or community/social networks fostered caregiver coping. Significant financial burdens due to cancer treatment, having to take unpaid leave from employment, remaining employed regardless of one’s circumstances, and lack of support from one’s health system, organizations, or community/social networks hindered caregiver coping. Our findings point to several policies that may ease caregiver burden and facilitate caregiver coping during childhood cancer treatment.

## 1. Introduction

It is estimated that approximately 9620 children under the age of 15 living in the United States (U.S.) will be diagnosed with cancer in 2024 [1]. The most recent data suggest that globally more than 275,000 children and adolescents were diagnosed with cancer in 2022 [2]. Due to advances in treatment, 85 percent of children will survive their diagnosis for five years or more [3]. Nevertheless, a childhood cancer diagnosis can result in severe stress and a sense of loss as families face tremendous disruptions to their daily life [4,5,6], often resulting in lasting psychosocial impact for patients and their families [7,8,9,10,11,12].

Sources of family stress include the trajectory to diagnosis [13]; the patient’s specific cancer diagnosis and prognosis [14,15]; the short- and long-term effects of treatment [14,15,16]; and lengthy hospital stays [17]. Additional caregiver stressors include managing their child’s needs in the hospital and at home [6,18,19], witnessing and participating in procedures, and changes in the patient’s appearance and quality of life [18,19].

Furthermore, the financial strain of out-of-pocket medical costs is a known factor that can add to the psychosocial burden of childhood cancer, disproportionally affecting those already struggling to make ends meet [20,21,22,23]. Families living in rural areas experience a higher risk of loss of social support and added financial burden from having to relocate to urban centers for specialized treatment [17,24,25,26]. In the U.S., Medicaid (insurance for low-income individuals and families) is available to those who qualify; however, coverage varies greatly by state in terms of eligibility and benefits [27].

The reduced ability for many caregivers to adhere to a regular work schedule while their child is undergoing treatment can also be a source of stress. The U.S. is currently the only “high-income” country that does not have a guaranteed national paid sick leave program [28,29]. Instead, parents of sick children must rely on policies enacted at the state and local level. Less than one in four (23%) U.S. workers had access to paid family leave in 2021 [29]. Research indicates that the combination of added expenses from cancer treatment and loss of income from work disruptions experienced by caregivers can pose significant financial and psychosocial ramifications for families, often well into survivorship [23].

Although findings are mixed, recent studies suggest the psychosocial impact of severe stress from childhood cancer can lead to significant experiences of trauma [6,30,31,32,33,34]. For instance, experiences with childhood cancer are associated with increased levels of anxiety and depression, impacting marital, familial, and social relationships [5,16], often persisting long after treatment has ended [30]. To mitigate the effects of childhood cancer, families employ a variety of coping mechanisms. Research has found that family functioning has a strong impact on mitigating (strong family cohesion) or worsening (poor family functioning) psychosocial outcomes for children with cancer, their siblings, and their caregivers [35].

To increase the understanding of why some families are able to adjust to their “new normal” [11] better than others, researchers have developed and employed an array of frameworks to identify key factors that impact a family’s ability or inability to adapt, largely centered around individual and family level factors [11,16,31,35,36,37,38,39,40,41,42,43]. For instance, the FAAR model [41,44] identifies three domains by which families cope with the experience of childhood cancer: appraisal-focused, problem-focused, and emotion-focused coping behaviors. Miedema et al. [11] modified the FAAR model by including additional coping strategies (e.g., alcohol misuse) indicating that not all coping behaviors are advantageous or helpful to families.

To the best of our knowledge, few studies have included the socioeconomic impacts of childhood cancer in their examination of family coping. In their systematic review of family functioning in the context of childhood cancer, Neugebauer and Mastergeorge [35] describe the lack of studies that have included financial stress as a stand-alone variable or have considered the impact financial stress may have on family relationship processes. We found only three empirical studies that describe the impact of socioeconomic factors when examining coping among families impacted by childhood cancer: Ochoa-Dominguez and colleagues [40] included financial impacts in their study of psychosocial wellbeing and coping strategies among 15 Hispanic parents; Patterson and colleagues [41] examined the impact of resource strains on coping behaviors, which included a brief mention of financial impacts; and Garge-Bouchard and colleagues explored the relationship between caregivers’ socio-demographic characteristics, including socioeconomic status, and the coping strategies used to adapt to childhood cancer, but they did not examine the added burden of costs associated with cancer treatment [45]. Additional gaps in research include missed opportunities to incorporate meso-level (organizational and community) factors (e.g., resources available through health systems, charitable institutions, or through a family’s social networks) when examining the ability of caregivers to adapt to their child’s cancer. The purpose of this article is to provide an in-depth examination of structural level (i.e., U.S. health care and leave policies) and meso-level (i.e., organizational and community) impacts on caregiver coping during the treatment phase of childhood cancer.

## 2. Materials and Methods

### 2.1. Design

We conducted a secondary thematic analysis of our comprehensive qualitative study examining family experiences with childhood cancer. Interviews emphasized narrative storytelling, in which study participants were provided with an opportunity to highlight what mattered most to them [46,47]. At the onset of each interview, participants were invited to describe the trajectory of their experiences as caregivers of a child diagnosed with cancer without interruption. This modified grounded theory approach [48] enabled the research team to examine emergent participant perspectives and understandings that were not originally anticipated [46]. Once the participant(s) had provided their narrative, the interviewer probed topics brought up by the participant(s) and asked questions from an a priori semi-structured interview guide (Supplementary Material A), developed from a review of the existing literature and consultation with an advisory group of clinicians, patient advocates, and caregivers of children who had experienced cancer. Interview questions focused on the process and perspectives of receiving the cancer diagnosis, experiences with treatment, the impact of cancer on the family (including financial impact, coping strategies, and social support), and survivorship transitions. Interview guide refinements were made as the study progressed and new themes were identified.

### 2.2. Recruitment of Participants, Ethical Considerations, Data Collection, and Study Participants

#### 2.2.1. Recruitment of Participants

A maximum variation sampling strategy was used to recruit study participants [46,49]. Participants were recruited through a letter from an oncologist, community outreach, and national social media campaigns. Moreover, special consideration was made to include socioeconomically diverse participants through outreach from a network of federally qualified health centers. Any family member who participated in the child’s care was invited to participate. If the patient was 16 or older at the time of the interview, they were also invited to participate. Families were eligible if they had a child diagnosed with cancer before the age of 15 who had completed curative treatment at least one year before their interview date.

We define coping as the “strategies a person/family uses to manage stressful life events” [50]. Caregivers are defined as family members or others (i.e., foster parents) who were responsible for the care of the cancer patient during their treatment.

#### 2.2.2. Ethical Considerations

Participation was voluntary and all participants received additional information about the study by phone before scheduling their interview. All participants had the opportunity to ask additional questions about the study and were provided with written informed consent prior to their participation in the study. The study was approved by the Institutional Review Board at Oregon Health & Science University (IRB: 00015946) on 14 April 2016. Quotes from study participants have been deidentified in order to protect their identity.

#### 2.2.3. Data Collection

Interviews were conducted between August 2018 and January 2020 by two interviewers with doctoral-level training in qualitative methodology (E.K. and V.C.). Interviews were recorded in either the participant’s home, a location of their choosing, or remotely via a secure platform. If more than one family member was present, interviews were conducted simultaneously. Interviews lasted 90 min to three hours. One USD 50 gift card was provided to each family for their participation.

#### 2.2.4. Study Participants

A total of 55 participants (33 mothers, 15 fathers, 1 grandparent, and 6 cancer survivors) representing 39 unique cancer cases were interviewed for the larger study. The following analysis focuses on 38 unique cases in which all 49 caregivers who were interviewed (33 mothers, 15 fathers, and 1 grandparent) were included. The original study included one patient who was interviewed without a caregiver present and was, therefore, excluded from this secondary analysis. Patient demographic characteristics and diagnosis are included in Table 1. Most caregivers interviewed were white females.

### 2.3. Data Analysis

With this secondary analysis, we explored new themes [51] to determine how structural and meso-level factors impact caregiver coping as they navigate childhood cancer treatment. Utilizing a modified grounded theory approach [48], as outlined in the Database of Individual Patient Experiences (DIPEx) analytical framework [46], five members of the original research team (V.C., E.K., K.P., L.C. and L.S.) independently dual-coded participant transcripts, focusing on the interpretations caregivers shared of their experiences that impacted coping, using inductive thematic analysis [52]. New codes were combined to create themes and subthemes using a constant comparison method [48]. Identified themes were deliberated among study team members using an iterative process until consensus was reached. An initial codebook was developed, and refinements were made as new themes and subthemes were identified. NVivo V.12 (QSR) was used to organize the data for analysis. The unit of analysis for this study was the child diagnosed with cancer. Our analysis focuses on the interpretations caregivers made of the impact structural and meso-level factors had on their ability to cope with childhood cancer.

## 3. Results

Our analysis revealed that caregivers experienced multiple and often intersecting structural and meso-level factors, both facilitating and impeding their ability to cope during the treatment phase of their child’s cancer trajectory. Caregivers described the financial impact of treatment, the toll their child’s treatment had on their ability to keep a regular work schedule, the impact of employment leave policies, and the role specific organizations and social support networks played in their ability to adjust to their child’s cancer diagnosis and treatment. Each category and resulting subthemes are described in detail below. Supplementary Material B presents a categorical framework of the themes and subthemes identified in our analysis.

### 3.1. Structural Factors

Caregivers discussed multiple factors that often combine in ways that make it difficult for families to adjust financially, such as struggling to pay for the out-of-pocket costs of cancer treatment while also having to take unpaid leave from work. Other families described how their circumstances (i.e., having Medicaid, insurance coverage with few out-of-pocket expenses or the ability to absorb the additional costs associated with treatment, and/or having flexible employers) helped them avoid significant financial hardship.

#### 3.1.1. Financial Burden Due to Costs of Care

Our findings indicate three major subthemes regarding added expenses due to the costs of their child’s cancer treatment, which included struggling to pay for treatment; receiving state medical (Medicaid) benefits; and having “gold standard” insurance with few out-of-pocket costs.

#### 3.1.2. Paying for Care Was Difficult

Caregivers discussed how paying for care was often challenging and, in some circumstances, led to bankruptcy or a considerable loss of savings. In many cases, the added financial strain associated with cancer treatment had a negative impact on the ability of caregivers to cope.

“*You’re sitting there looking at all these medical bills… Like my stress with money is through the roof…that’s a lot of money, that’s $20,000, and to this day, we don’t have $20,000 just lying around. So, it’s a whole new level of stress…we ended up having to do a bankruptcy” [Participant 21]. Participant 07 shared, “We had quite a nice savings before we started this whole thing. And we blew through all of our money and…we literally had to borrow money from my family…we blew through $100,000…And just watching each month as we slowly lost everything.” Participant 22 recalled: “You’re kind of flying solo the second year going, ‘I am still broke. I still do not have my finances in order.’ Savings were gone*.”

#### 3.1.3. Receiving State Medical Coverage (Medicaid)

Some caregivers talked about qualifying for and obtaining state health care coverage (Medicaid) for their child, which, in most cases, eliminated out-of-pocket expenses related to their treatment. For those who had Medicaid coverage, not having to incur debt due to the costs of care was seen as a “huge relief”, as Participant 30 described: “*we don’t have copays for any medications, any therapy, for anything—nothing…it’s just such a stress relief not to have that*”. For Participant 09, moving to another state for a new job resulted in state-sponsored medical coverage for their daughter: “*The state we were in for our first hospital [stay] had this amazing law that she would be covered by Medicaid. They gave us a card and they paid for everything that entire year as long as we were residents of the state…and it was amazing*”. Participant 12 described finding out that they could qualify for Medicaid coverage: *“… I had no idea about this before—but if you’re a child, and you make under a certain amount of money they cover it…so all of a sudden, we didn’t have to worry about our bills anymore. That was a huge relief*”.

#### 3.1.4. Having “Gold Standard” Health Insurance and/or Being Able to Pay Out of Pocket Expenses without Hardship

A few families reported having “gold standard” health insurance in which they paid very little out-of-pocket for medical expenses and/or they had the ability to absorb the impact of additional medical bills. For these caregivers, rather than the added stress of financial instability, they viewed their financial situation in a positive light, feeling fortunate that they did not have to worry about the financial impact their child’s care would have on their family. Participant 05 talked about feeling fortunate that, because of their situation, they did not incur any debt during their child’s cancer treatment: “*So, for us, we’re very fortunate, economically. I would say the impact is minimal. Our health insurance is excellent, so we didn’t have any issues with paying for medical care. My husband’s job allowed him plenty of time off. I was a stay-at-home mom so I didn’t have to miss work…so I really can’t complain about the economic side of things*”. For Participant 24, “*The other thing that made it easier—is that we’re financially comfortable and we have insurance. Those two things make a big, big difference…we could just do it, and it was okay*”.

#### 3.1.5. The Ramifications of an Unpaid National Family Leave Program (FMLA)

In order to take time off work to care for their child, caregivers recalled having to navigate complex federal and state policies. The federal Family Medical Leave Act (FMLA) currently guarantees eligible employees *unpaid* leave for up to 12 weeks per year [53]. Our findings identified three major subthemes regarding the impact of FMLA in its current form: struggling to make ends meet while taking unpaid time off work to care for their child; caring for their child but still needing to work to maintain an income regardless of their circumstances; and having employers that provided them with paid time off or having coworkers that contributed their accrued sick time, or simply covered their work shifts.

#### 3.1.6. Struggling to Make Ends Meet While Not Being Able to Work

Caregivers talked about making the difficult decision between keeping their jobs and their income or taking unpaid leave to care for their child. For many, the added financial toll of not having a regular income led to an increase in stress, thus impacting their ability to cope with their child’s diagnosis and treatment. Most often, it was the patient’s mother who stepped back from regular employment. Participant 01 recalled the significance of her family’s loss of income: “*We almost lost the house because I wasn’t working. I had to stay home with him… Afterwards, when he was a year off of treatment, I did go back to work to try to pay down some of the medical debt from the following years*”. For Participant 22, their financial struggles were due to a combination of their child’s medical bills and having to miss work to stay at the hospital with their child: “*It was absolute hell…You do not know how you are going to pay for it. You just know that you have to do it. We took out personal lines of credit and paid that off. Yeah, the financial was a huge hit. I could not work a lot. My job was so understanding…but you still do not get paid. Your bills are still coming in. Now you are getting more bills because now you are in the hospital*”. Participant 08 shared the long process of paying off her family’s debt due to taking a leave of absence from work: “*We’re still trying to get caught up. Well, because like with me I had to be with [Patient]. And so, my job’s gone. I’m like credit cards in debt…I mean it sucks that you can’t—that there’s no insurance that helps cover it if you have to leave work or anything like that…[I’m] still trying to dig out of it*”.

#### 3.1.7. Caring for Their Child but Still Needing to Work

Some caregivers felt fortunate that their employers were somewhat flexible regarding their work schedule, but, nonetheless, it was expected that they completed their work if they were to continue to receive a paycheck. For these caregivers, they found it difficult to manage caring for their child while maintaining their work-related expectations. This added to their overall stress as they had little time that was not accounted for. For Participant 03, although his employer was flexible, it was still difficult to manage both work and taking care of his daughter: “*Cancer breaks people financially. I was broke. …I could come to work late. He [employer] would accept it, as long as I worked 32 h a week, which is an ungodly amount of hours of work [while] dealing with everything else. He didn’t always get that. I did what I could*”. Participant 22 spoke of the difficulty of working and caring for his daughter: “*I remember driving to work going I just pray we do not have any hospital stays. My bosses have dealt with me long enough…then four hours later, I was going ‘she just got admitted. I have to leave early*’”.

#### 3.1.8. Getting Paid Leave through Their Employer or through Coverage from Coworkers

A few caregivers recalled feeling lucky that they were in a position in which they or their partner could take leave from work and still get paid. In some instances, their employers were supportive and, in other cases, coworkers donated their accrued sick time or simply stepped in to cover work shifts. Participant 04 discussed how he felt fortunate because his wife works for her family’s business: “*We’re sort of lucky in a lot of ways…our life is set up that we can deal with this. …I’ve been the primary worker. You know, [Patient’s] mom works from home, …she works with her family, it’s very easy to say, ‘Okay, I’m not going to be able to work today because of [Patient],’ and they’d be totally, ‘It doesn’t matter.’ …[Patient’s] mom could deal with it, and I could continue to make sure that we have money*”. Participant 33 talked about how her husband’s supervisor and coworkers were supportive: “*His captain…came and told him not to even think about coming back to work until this was all done, which was six months…and he just completely released my husband from work. And the guys that he worked closer with, they covered his shifts. Like it was such a gift…for him to not to have to worry about going to work, that was huge*”.

### 3.2. Meso-Level Factors

Organizational and community level factors, such as services provided by a health system, support from an organization, or one’s social network, can play an important role in helping mitigate some of the stressors associated with childhood cancer. Caregivers who reported having received such support often described it as a financial and/or an emotional lifeline. Not all participants received such support, however.

#### 3.2.1. Support from Hospital Staff and/or Charity Organizations

Few caregivers described being offered mental health support from their health system during their child’s treatment, as Participant 05 lamented: “*that probably has a lot to do with the fact that the medical people are doing the medical side of things and there’s not the whole psychological emotional support that there could be*”. Participant 15 recalled, “*I almost wish that they made you go to some kind of support group…I have really bad PTSD from it, from the whole experience*”. Our findings indicate, however, that hospital and clinic staff often provided a sense of comfort during their child’s treatment, which helped foster a positive outlook among caregivers. In addition, several caregivers recalled feeling fortunate that their social workers, who were assigned to them by their child’s treatment center, provided them with organizational contacts who could assist with the financial burden. Caregivers who were unable to benefit from such resources described frustration when finding out about programs too late to take advantage of them or because their health system did not have the resources to assist with such support.

#### 3.2.2. Feeling Cared for by Hospital Staff

Several caregivers spoke of enduring extended hospital stays during their child’s treatment, which were often emotionally and financially difficult. Yet, many caregivers also recalled feeling genuinely cared for by people who worked or volunteered at the hospital. This made all the difference to them as they went through, what was for many, the most stressful time of their lives. Caregivers talked about hospital staff members who worked hard to make the experience as positive as possible. Participant 32 described it this way: “*One of the best things that happened when we were in the hospital was meeting child life specialists, which I didn’t even know existed. They are just amazing people that come in and help distract your children and just shower them with love. They helped us decorate his room in the hospital, because 35 days was a long time being in this one room. But they made it so it didn’t seem scary*”. Participant 33 talked about feeling that the nurses who cared for her son were passionate about their work: “*Something that they did on that floor, …it was so many laps around was a mile. …by the time we finished treatment, he ended up doing a 5K in his walker all the way around there…and the nurses celebrated him, like made a finish line for him to cross through. And like that boy received more love in that time, and that’s such a silver lining of it*”.

#### 3.2.3. Finding Out about Opportunities That Helped Families Financially

Caregivers described how connections to social workers, who provided them with information about opportunities for assistance, made a significant impact on their emotional wellbeing. Others spoke of being aware of charitable organizations that they found to be helpful. Participant 17 explained how the social workers at the hospital helped them secure resources so they could focus on their child: “*The social workers were amazing. They helped with any bills we had. …You have the financial stress on top of it, oh my god…how are we going to going to do this? They come to you and they say ‘okay do not worry about this month you know we are going to take care of all’…and it gives you time to worry about your child*”. For Participant 19, “*there was a lot of traveling…it was pretty rough, …then we started learning about organizations that help with stuff, so we’ve had our plane tickets completely paid for. …Once we learned about that, it was like a whole game changer. It kind of made everything a lot easier*”.

#### 3.2.4. Not Receiving Help from Their Health System and Missing Out on Resources

Other caregivers recalled the frustration of not knowing they had access to a social worker as part of their care team, while others simply did not have this resource available to them. The lack of a connection to someone who is knowledgeable about different resources and missing out on the potential for help was viewed as having an adverse impact on their ability to cope. Participant 22 shared how they would have benefited from such support: “*We did not even know that there was a social worker assigned to kids. I had no clue. We did not know that we had an outlet there to talk to him. If there were any programs or anything that could have helped in any way… I think, would be important for families to know there is support. There is help…we were like, ’Oh wait. What?’ …I think if we would have had that right away, maybe we would have had avenues for some type of counseling, group sessions, or something like that*”. Participant 13 described finding out too late that there was help available: “*It wasn’t until a couple of weeks ago that people were saying ‘Oh, did you know the Cancer Society will help cover lodging?’ No,…I didn’t have any of that information. We were using all of our miles, all of our friends’ air miles and whatever we could come up with and someone says ‘Oh, well, you know you could try this Angel Flight thing?’ I’m like ‘what’s Angel Flight?’ Well, you’ve got to go through your social worker. It’s like ‘Oh, well, that would have been helpful.’*” Participant 14 explained that they wish they were given more information about resources that were available to them: “*There was not and there still is not any…manual for parents. Like…some sort of—‘These are resources in one book. Here is the food stamp office. Here is the Medicaid office. Here is Northwest Sarcoma Foundation’s information. Here is Cancer First. Here are all of the things you need to be able to survive.’ Not only are you trying to survive at the hospital, but there is whole other life somewhere else that you left to go do this*”.

#### 3.2.5. The Impact of One’s Social Network on Coping

Several caregivers commented that they received support in several different ways through their extended families, friendships, social groups, and from other affiliations. Some families described support through “Go Fund Me” campaigns, help with meals and household chores, or gift cards for gas and groceries. Others talked about feeling supported when friends and family visited, provided comfort, or helped care for their child’s siblings. Caregivers recalled connecting with other “cancer families” and how these connections made a positive difference. Occasionally, families reported not receiving the type of help they needed or that support from others lasted only a short while. For these families, lack of support added to the emotional burden they experienced during their child’s treatment.

#### 3.2.6. Receiving Financial Support

Some caregivers described receiving financial support from their social network (e.g., friends, extended family, and community) through fundraisers such as “Go Fund Me” campaigns or through other fundraising mechanisms. Such support was viewed as helping to reduce financial stress. Participant 01 recalled receiving help from their “AA family”: “*A lot of it was our other family, our AA family because they did pancake breakfasts. When people were beginning to find out, everybody donated. There was one person who opened an account for [Patient], and everybody donated that could. And it ended up paying his medical bills for the first year. It was over $3000, which was his maximum out of pocket deductible for that year*”. Participant 13 mentioned how their “*church family has been supportive. I mean the day we were leaving, a guy from church sent his son to the door. He said there’s an envelope. I opened it. It was a check for $10,000*”.

#### 3.2.7. Receiving Emotional Support Form Extended Family, Friends, and Members of Their Community

Caregivers who received support outside of their nuclear family recalled feeling fortunate that some of the pressure associated with their child’s treatment had been reduced, often leading to increased resiliency. Emotional support was viewed as a key aspect of their ability to cope and adapt to their new circumstances. For many, support came from extended family and friends. Participant 24 recalled having a “*really robust support system…we basically outsourced everything we could and dropped everything we could drop*”. Participant 29 talked about the help they received from their church: “*During that time, we were very involved with the church…and they came up to the hospital a lot, they had prayer groups for us”.* Participant 24 shared how she stayed connected with friends: *“We had a lot of local families, so we had a really robust support system…*”.

#### 3.2.8. Connecting with Other “Cancer Families” Who Were in a Similar Situation

In some instances, caregivers spoke of developing strong bonds with other families they met at the hospital. In these cases, caregivers often felt an immediate connection because of their shared circumstances. Caregivers talked about receiving help and support during challenging times and that knowing others could relate to what they were going through made all the difference. Participant 20 described it this way: “*there’s this comradery of the group of people whose families [have] experienced this*”. Participant 32 shared her experience of connecting with another parent: “*I started talking to a dad that was in [the craft room]. He actually said, ‘[Is] this is your first day?’ and I said, ‘Yes.’ And he said, ‘It will be okay.’ And he basically told me how his child was in there again, for stem cell transplant. …He was one of my very first contacts there. … It was just like a light in the dark because we were scared and really didn’t know like was this a death sentence. …It was such a comfort to talk to somebody who knew what we were going through, exactly when we were going through it”.* For Participant 22, being able to relate to other families going through a similar experience was important: “*Seeing her go to school with normal kids who were healthy was fine. But seeing her with the other kids who were also bald, or may have a tube up their nose, or whatever; that was just amazing. They could all be themselves, and nobody is going to judge them. That was really important for us. Then, we got to connect with other families who were going through either the same or similar. You can just relate*”.

#### 3.2.9. Not Receiving the Social Support They Needed

In other cases, participants recalled receiving support in the beginning of their child’s cancer treatment but that it did not always last very long. For some, this came as a surprise, while others had been warned this would happen. Loss of support was difficult both emotionally and in terms of having to fill in the gaps where help was lost, such as meal preparation. Participant 22 lamented: “*You lose family and friends, as sad as that is*”. Participant 32 discussed her family’s experience after the shock of their son’s diagnosis wore off: “*Our experience with the people, like our friends and family and stuff, they were wonderful, especially that first month when you really need support. My husband and I noticed that our group of friends kind of died off after that. It was like they didn’t know how to support us or what to say to us, so they just left. And that was another cancer trauma I guess that you’d say that was added to us. Because we kind of just felt abandoned by a lot of people that we felt would always be there*”. Participant 25 shared: “*Everybody comes in and helps you in the beginning…then, it kind of goes away. You feel that from your community. You feel that from your family…you also feel it from the doctors. I don’t think people really realize how difficult that is*”.

A few participants recalled wishing they had been able to connect with other families at the hospital. For Participant 19, because her son received his treatment as an out-patient, it was difficult to connect with other caregivers who were going through something similar: “*What I wished I’d had more of the emotional support, I think, being a parent, being a mom. Because I never talked to any of the moms that were going through it with their kids. You know, especially, that first set of chemo. Like I said, we were in these little rooms and the door was shut and the nurses would come in and out. But I never saw any other parents…I almost wish somebody would’ve said, ‘Here…this is part of your treatment plan. Mom and Dad need to go and meet with these people once a week and talk to them about what’s going on*’”.

In sum, our findings suggest that added financial burden and lack of social support (from both organizations and social networks) can have significant impacts on the emotional wellbeing of caregivers, ultimately impacting their ability to cope. These stressors often add to an already stressful situation in which caregivers must navigate their child’s specific cancer diagnosis and prognosis, the effects of treatment, disruptions to family schedules due to long treatment protocols and lengthy hospital stays, and the difficulties of managing their child’s needs in the hospital and at home. These findings demonstrate the importance of looking beyond individual-level coping structures when examining why some families are able to adjust and thrive while others struggle during their experiences with childhood cancer.

## 4. Discussion

By utilizing a sociological framework focusing on structural and meso-level factors, our paper adds to the body of research examining factors that enhance and impede the ability of caregivers to cope and adapt as they adjust to their child’s cancer diagnosis and treatment. Our analysis reveals that families experience multiple structural and meso-level factors simultaneously, often having a compounding effect on their ability to cope with the challenges and stressors of having a child with cancer. We found that the U.S. privatized health care model, which includes variable deductibles and out-of-pocket expenses, leaves many caregivers struggling to cover the costs associated with their child’s treatment. This is especially the case for families who do not qualify for state sponsored medical coverage (Medicaid) but lack the resources to absorb the added medical costs associated with cancer. For these families, the added financial impact adds an additional layer of stress, leaving caregivers vulnerable to increased feelings of distress, thus hindering their ability to cope. This finding is similar to that of other studies that have included financial impacts when examining caregiver and family coping [40,41] as well as studies examining the financial toxicity of cancer treatment among adult cancer patients in which added financial strain increases the likelihood of anxiety and emotional distress, leading to diminished health-related quality of life [54]. We also found that families described having access to Medicaid as “*a huge relief*”, while having “gold standard” health insurance and/or the ability to absorb the additional costs of their child’s treatment was viewed as enhancing a family’s ability to cope. As one caregiver stated, “*we could just do it, and it was okay*”.

Our analysis also revealed that FMLA leaves many families struggling to care for their child with cancer, who may experience long hospital stays and/or may need to stay home from school or daycare for extended periods of time. Several caregivers reported they had to choose between remaining employed or caring for their child soon after receiving the devastating news of their child’s diagnosis. Often, it was the mother who took on caring for their child with cancer, which frequently caused disruptions in employment. Other caregivers reported juggling both work and caring for their child and that the experience was overwhelming. A few caregivers reported receiving paid leave or that their coworkers stepped in to help. In these instances, not having to work was seen as a “*gift*”, greatly contributing to their emotional wellbeing. Our findings are consistent with the broader literature examining the financial toxicity associated with childhood cancer and the resulting impacts on families [20,23].

Meso-level factors played an important role in the ability of caregivers to cope with their child’s treatment. Caregivers talked about feeling supported by hospital staff who were often their source of comfort during a challenging time in their lives. Receiving vital information or being able to secure additional resources that helped mitigate the financial impact of their child’s care played an important role in the emotional wellbeing of some caregivers. Our analysis indicates, however, that a significant number of families who may qualify for such resources are unaware that they exist.

Support from one’s social network or community, either through “Go Fund Me” campaigns, helping with daily tasks, or providing emotional comfort, was viewed as providing a lifeline, helping caregivers cope as they dealt with their child’s treatment. Caregivers also described the strong bonds they developed with other “cancer families” they met at the hospital. These relationships provided caregivers with important reinforcements, such as receiving insight as to what to expect from various cancer treatments or receiving encouragement that there was a “*light in the dark*”. Other caregivers, however, spoke of feeling abandoned by those they thought would be there for them or found they we unable to connect with other families at the hospital.

Our findings also point to organizational and national policies that can be implemented to help caregivers as they navigate the daunting and often overwhelming challenges associated with childhood cancer diagnosis and treatment. To mitigate some of the impacts associated with childhood cancer, we recommend that cancer centers implement mental health support programs for caregivers and siblings throughout the cancer trajectory [19,55,56] and designate a social worker/child life specialist to each family across health systems with standardized supportive care [55]. Such programs should include informing caregivers about the resources and services for which they qualify, providing them with needed information that they may not have the time or skillset to locate on their own. Further research is needed, however, to determine the most effective implementation strategies for delivering such resources to families in need of support.

At the national level, policies should be developed to help families facing financial hardships. For children living in the U.S., one option would be to allow children diagnosed with cancer to qualify for secondary insurance through Medicaid, which could help offset the financial impact of treatment. Furthermore, the shortcomings of leave policies such as FMLA point to the need for universal guaranteed pay during a medical crisis such as childhood cancer. As written, FMLA covers only larger workplaces and limits eligibility to workers who meet the requirements of minimum tenure and hours worked to obtain job security during a leave of absence. Currently, in the U.S., only 14 states, the District of Columbia (D.C.), and 20 jurisdictions have passed laws requiring covered employers to provide eligible employees paid time off for their own illness or to care for their sick children [29]. Long treatment schedules, including unplanned hospital stays, often result in reduced work schedules, ultimately affecting financial stability for many caregivers. Programs that foster stability among families caring for a sick child have been shown to mitigate the burden of cancer and lessen the related stressors experienced by caregivers, which, in turn, can lead to increased resiliency and improved patient outcomes [10,56,57].

### Limitations and Future Directions

Providing caregivers with the opportunity to describe their experiences with childhood cancer, focusing on what mattered most to them, enabled caregivers to recall factors that both fostered and hindered their ability to cope throughout their child’s cancer trajectory. Limitations to this methodology include the potential for recall bias. Including eligible cancer survivors who wished to participate in a single interview with their caregivers (*n* = 5) may have impacted how caregivers described their narrative. Furthermore, the demographics of our study participants did not allow for analysis of the impact of other structural factors (racism, socioeconomic status prior to diagnosis, and geographic locality) on caregivers’ ability to cope. Future research is needed to examine the full range of structural and meso-level factors that foster and impede caregiver coping during the treatment of childhood cancer. It is also important that future research examines how structural and meso-level factors impact experiences during survivorship, as the completion of treatment does not necessarily mean an end to the stressors associated with cancer and treatment. Such research should include an examination of the lasting impact of stepping back from employment to care for one’s child after a cancer diagnosis. Moreover, an international comparison of health care models, leave policies, and access to organizational and community resources should be undertaken to determine similarities and differences in how national health and leave policies and organizational-level factors impact caregiver coping with childhood cancer.

## 5. Conclusions

Few studies have focused on the impact of structural (i.e., health care delivery and leave policies) and meso-level (i.e., organizational and community) factors when examining caregiver coping with childhood cancer. Findings from our in-depth analysis demonstrate that institutional, organizational, and social support factors play an important role in a caregiver’s ability to cope with their child’s diagnosis and treatment. Our research also points to health system and national policy changes that may foster resiliency and improve quality of life, particularly for those coming from lower socioeconomic backgrounds. Implications for clinical practice include helping families locate resources that may be available to them and developing programs specifically aimed at improving the psychosocial experiences of caregivers. By focusing on factors beyond individual-level strategies of coping, our research points to the need for further examinations of structural and meso-level factors that may impact a caregiver’s ability to adapt and cope as they face the challenges associated with childhood cancer.

## Figures and Tables

**Table 1:** Patient characteristics.

Participant ID	Diagnosis	Patient’s Sex	Patient’s Age at Diagnosis	Patient’s Race/Ethnicity	Insurance during Treatment	Geographic Location	Caregiver (Participant) Relationship to Patient (N = 49)
01	Leukemia	Male	3 years	White	Medicaid	Urban	Mother, Father
02	Embryonal Rhabdomyosarcoma	Male	14 years	White	Private	Urban	Father
03	Leukemia (ALL)	Female	14 years	White	Medicaid	Urban	Father
04	Leukemia (ALL)	Female	6 years	White	Private	Urban	Mother, Father
05	Wilms tumor	Female	4 years	White	Private	Urban	Mother
06	Leukemia (ALL)	Female	8 months	White	Medicaid	Urban	Mother, Grandmother
07	Leukemia (ALL)	Male	2.5 years	Multiracial	Private	Urban	Mother
08	Leukemia (AML)	Male	16 months	Black	Medicaid	Urban	Mother
09	Leukemia (ALL)	Female	14 months	Multiracial	Private	Urban	Mother, Father
10	Leukemia (ALL)	Male	5 years	Multiracial/ Hispanic	Medicaid	Urban	Father
11	Hodgkin’s Lymphoma	Male	8 years	White	Medicaid	Urban	Mother
12	Leukemia (ALL)	Male	3 years	White	Private	Frontier	Mother, Father
13	Medulloepithelioma	Female	9 years	White	Medicaid	Frontier	Mother, Father
14	Ewing Sarcoma	Female	6 years	White	Medicaid	Frontier	Mother
15	Leukemia (ALL)	Female	4 years	White/ Hispanic	Medicaid	Urban	Mother
16	Leukemia (ALL)	Male	2.5 years	White	Not reported	Urban	Mother
17	Ovarian cancer	Female	10 years	White	Not reported	Urban	Mother, Father
18	Retinoblastoma	Male	3 months	White	Not reported	Urban	Mother
19	Hodgkins Lymphoma	Male	8 years	White	Public health plan (CAPH)	Urban	Mother
20	Wilms tumor	Female	2.5 years	White	Not reported	Urban	Mother, Father
21	Wilms tumor	Male	2 years	White	Private	Urban	Mother, Father
22	Lymphoma (NHL)	Female	4 years	White	Medicaid	Urban	Mother, Father
23	Retinoblastoma	Female	18 months	White	Not reported	Rural	Mother
24	Rhabdomysarcoma	Male	4 years	White	Private	Urban	Mother
25	Leukemia	Female	13 years	White	Private/Medicaid	Urban	Mother
26	Leukemia	Female	4 years	White	Private	Urban	Mother
27	Wilms tumor	Male	4.5 years	White	Other	Urban	Mother
28	Neuroblastoma	Female	4.5 years	White	Not reported	Urban	Mother
29	Ewing Sarcoma	Female	13 years	White	Private	Urban	Mother
30	Ewing Sarcoma	Male	6 years	Multiracial	Medicaid	Urban	Mother
31	Leukemia	Male	2.5 years	White	Private/Medicaid	Urban	Mother
32	Leukemia (ALL)	Male	3 years	Other	Other	Rural	Mother
33	Leukemia (AML)	Male	4 months	White	Private	Rural	Mother
34	Rhabdomyosarcoma	Female	13 years	Not reported	Not reported	Urban	Father
35	Lymphoma	Male	11 years	White	Private/Medicaid	Rural	Father
36	Neuroblastoma	Female	15 months	Multiracial/Hispanic	Private	Urban	Mother, Father
37	Leukemia	Male	5 years	Multiracial/Hispanic	Private/other	Urban	Mother
38	Chronic Myeloid Leukemia	Female	11 years	White	Private	Urban	Mother

## Data Availability

Deidentified transcripts are available from Oregon Health & Science University upon request through a Data Use Agreement.

## Supplementary Materials

The following supporting information can be downloaded at: https://www.mdpi.com/article/10.3390/ijerph21070907/s1: A semi-structured interview guide (Supplementary Material A) and a categorical framework of the themes and subthemes identified in our analysis (Supplementary Material B).
