# Supplementary material for: The Impact of Structural and Meso-Level Factors on Caregiver Coping Abilities When Supporting a Child with Cancer: A Qualitative Study
Source: Int J Environ Res Public Health. 2024 Jul 11;21(7):907. doi: 10.3390/ijerph21070907 (PMC11277199; doi:10.3390/ijerph21070907)
Supplement: Supplementary file 1 [file ijerph-21-00907-s001.zip › Supplementary Material A Interview Guide.pdf]

Childhood Cancer Interview Guide (Parent/Guardian)

- *Show participants website and explain why we are doing this….*
- *Remind participants to share the whole story - we want to hear all of it, the good, bad, etc. (all emotions are safe to share)*
- *We can stop and take a break at any time, or continue on another day*
- *I might not say yes or uh huh- I am still listening but don't want that to be on recording*
- *You will have opportunity to review your transcript and remove any items you don't want included.*

Interview Questions

1. Why did you decide to participate in this study? (either ask this here or at the end….)
2. Can you start by telling me the story of your child's cancer journey? (Feel free to start wherever you would like and to take as much or as little time as you would like. We will follow-up with specific questions when you are done, but first want to give you a chance to share your story with us).

Diagnosis

3. When did you first start noticing symptoms or suspect that something was wrong?
4. Can you walk me through the process of getting a diagnosis?
    a. How long did it take until you received an official diagnosis?
    b. Do you remember the conversation with your doctors about your child's diagnosis? If yes, can you tell me about this?
        i. What do you think could have gone differently or been done better?

Clinical trials

5. Were you offered the opportunity to take part in a clinical trial at any point during your child's treatment?
    a. **If yes**, can you walk me through what you remember about that process?
        i. **When** were you approached about participating in a clinical trial?
        ii. What was your understanding of the **purpose** of the trial?
        iii. Did you feel like you had **enough information** to decide whether to enroll your child on the trial?
        iv. Did you feel like you had **enough time** to make this decision?
        v. Do you recall the **consent process**? If yes, is there anything you wish had been done differently?
        vi. Did you **involve your child** in any of the decisions about treatment and/or whether to take part in a clinical trial?
        vii. Did you decide to take part? Why or why not?
        viii. If yes, did you ever receive the results of the study?
        ix. If yes, can you tell me about some of the good parts about taking part in a clinical trial? What about the bad parts?
6. Do you have any recommendations of ways to improve awareness, understanding or enrollment in clinical trials? (*optional*)

Treatment

7. Now I would like to ask you some questions about the treatment process. Can you walk me through what that was like for you and your child?
    a. **Where** did you receive your treatment? Did you have to travel for treatment? If yes, how far? How long did treatment last?
    b. Can you tell me about what it was **like to spend time at the hospital**/care facility?
        i. Did you get to know other families? (optional probe)
8. Did your child experience any side effects from treatment? If yes, what was most challenging?

a. What strategies did you use to manage the effects child's treatment (both physical and psychological)
b. What about at **home**? How did you cope/manage that?

9. What was it like to be at home with your child during treatment?
    a. Are there things that you did at home to support your child?
    b. What was the most challenging thing about being at home during treatment? (*optional*)

Medical care

10. In general, how do you feel about the medical care you received? Is there anything you wish had been different?
11. How accessible/responsive were your child's care providers to your questions?
12. Did your child's **care go according to plan** or was there anything that surprised you or that you weren't expecting?
13. What was the **impact** of your child's medical care on the **rest of your family**? Do you have other children? If so, what was this experience like for them? What did you do to try to create some sort of **normalcy at home**?
14. Were you **offered any services** during this time, such as **emotional support** or counseling for you or anyone else in your family?

Finding information

15. Did you seek any additional information about your child's treatment?
    a. How did you find answers to questions that you had about diagnosis and treatment?
    b. Were there people or resources that were particularly helpful to you? If yes, can you tell me about those? (e.g. internet books, handouts; social media; support groups; hospital or other resources at school; chaplain; organizations like CCA or make a wish)
16. Are there things you wish you had known about your child's treatment that would have made things easier?
    b. Are there any additional resources or sources of information that would have been helpful during diagnosis and treatment?
17. While researchers have not been able to definitively point to the reasons children get cancer, some people have discussed **wanting to know the cause** of *their* child's cancer. After your child's diagnosis, is this something you wondered about? What did you do? What resources did you use?

Hardest thing about diagnosis and treatment

18. Looking back, what was the hardest part about the process of diagnosis and treatment for your child? What about for you and the rest of the family?

Impact on Family

19. Thinking back on your child's cancer journey, what is the biggest way that it has impacted *your life* <u>*or*</u> *your family's life?*
    a. What about your relationship with your child? (possible probe: Did it change the ways you parent?)
    b. In what ways did it impact your relationships with others (e.g. spouse, other children, friends, extended family)?
    c. Did it impact any major decisions that you or your family made? (*optional*)
    d. In what ways did your child's experiences with cancer impact you or your family **financially**? Did it impact your job?
20. What strategies did you use to cope with the impacts on your family?
    a. Can you think of things that would have helped make things easier?

Life after treatment

Now I would like to ask you some questions about transitioning back to daily life after treatment.

21. What was it like for your child to finish the treatment process?
    a. What was the best part for your child about finishing treatment? What was the hardest part?
    b. Can you tell me what it was like for them to return to school?
        i. What are the biggest challenges (e.g. friends, cognitive impacts etc.)?
        ii. Is there anything that could have made this easier?
22. What was it like for *you* after the treatment process was complete?
23. What type of plan was established for ongoing medical care?
    a. If your child is sick or has a medical problem, who would you go to? (What type of provider)
24. How is your child's health these days? Does he/she experience any ongoing side-effects as a result of their cancer or cancer treatment?
25. What is your understanding of the **long-term** impacts, if any, of your child's **cancer or cancer treatment**?
    a. Did you feel fully informed and aware of these **long-term** impacts as your child was going through therapy?
    b. Is there anything you wish you would have known? Looking back, is there anything you would have done differently?

Messages for others

26. Why did you decide to participate in this study? (if didn't ask at the beginning….)
27. What is the most important thing you would tell a parent whose child has just been diagnosis with cancer? What would you tell a child who has just been diagnosed?
28. Are there things you would like to share with extended family members, friends, or others about how best to support children and families throughout their treatment and through survivorship?
29. Are there things you would like to share with health care professionals about how best to support children and families throughout their treatment and through survivorship?
30. Are there other aspects of your child's cancer journey or the impact on your life that we didn't ask you about that you would like to share with us?
