# Supplementary material for: The Impact of Structural and Meso-Level Factors on Caregiver Coping Abilities When Supporting a Child with Cancer: A Qualitative Study
Source: Int J Environ Res Public Health. 2024 Jul 11;21(7):907. doi: 10.3390/ijerph21070907 (PMC11277199; doi:10.3390/ijerph21070907)
Supplement: Supplementary file 1 [file ijerph-21-00907-s001.zip › Supplementary Material B Analysis Framework.pdf]

Supplementary File B: Categorical framework for understanding factors that impact caregiver coping during childhood cancer treatment

Theme	Category	Sub-theme	Code	Representative Quotes
Structural factors	Financial impact of childhood cancer	Financial burden due to costs of cancer treatment	Paying for care was difficult	"So, you know it's five years ago, but yet we're still sort of catching up on things. And, you know, figuring out our financial plan…planning each year, because he has an echocardiogram every year now. I have to plan for that every year now until forever. …So, yeah, it's, you know, huge financial. [19]
			State Medicaid insurance helped	"I was kind of blessed that she was on the Oregon Health Plan…I never had to pay a dime except for over the counter things…some of her medicines, I looked at how much they cost. They were like $300 upward for one round of it, and a lot of times I had to have two, three rounds for her." [06]
			Costs associated with care were not considered a financial hardship	"It was a private clinic but they have associations with the hospitals in Seattle. And we ended up paying very little. I mean what the insurance company didn't cover they basically waived." [02]
		Financial impact of needing to take leave from work to care for their child	Struggling to make ends meet while not being able to work	"If you already have a child going through treatment, but then to lose your rock...not only is your child sick at this point, but now you can't work…and yes, I have a short-term disability, but it does not cover you if your child is sick; it only covers if you're sick." [21]
			Having a flexible employer, but still needing to work	Were you able to take some family medical leave? Patient's Father: "They wanted me to do that. And I said 'that would actually not be helpful, because I mean it's like so my salary stops, you know that, right? So how does that actually help me?" [02]
			Obtaining paid leave from their employer or coverage from coworkers	"My mom worked at fish and game. So, a lot of state employees put in their leave to make sure mom had a paycheck." [14]

Meso-level factors	Support from the health system and/or charities	Information about resources	Getting assistance	"There were certain places that helped with our rent…a lot of the social workers up at the hospitals would have different places that they would contact that would help. They helped with gas. They helped get our wood for our fire. There were certain places that, you know, helped keep the roof over my kids' head. Got food boxes and stuff like that because it's definitely a shock to your financial stability." [08]
			Not knowing about opportunities for support	'I reached out to [social worker], I think, maybe once…. I know, for me, we're not people who say we need help…we're not ones who say 'we're struggling or we need help or how can we maybe work this out?' So maybe someone just checking in and asking like, 'Hey.' At least there's an option…to say, 'You know, yeah. Actually, if we could have some resources on this or that.'" [16]
		Emotional support from hospital staff	Receiving emotional support	"I think it was that I had a good team to help me through the process. I think it worked the best they could at that time. They were constantly checking on us. There would be times where the doctors would call us at home to make sure everything was okay." [11]
	Support from one's family or social network		Receiving financial support from family or social network	"[Patient's] Father's brother set up a fund that—well, he climbed a mountain on his bike and got a lot of people to contribute to that. And so, we did get a lot of help from people from that." [07]
			Receiving emotional support from family or social network	"My mom went to pretty much almost every appointment with me, and that was like huge because my mom does not like hospitals. So for me, that was support because that was a companion, for me…she actually came and stayed in the hospital with us, too. So she would kind of be our food go-getter, let me take breaks...just kind of help filling in the gaps." [16]

	Connecting with other “cancer families”	There's this comradery of the group of people whose families, or kids, or someone who has experienced this. [20]
	Not getting social support	“It seems like even with family support and everything, you have that high where everybody wants to help. Then there is that low where it is like, ‘oh you guys are still going through this?’ You lose family and friends, as sad as that is.” [28]
